# Influence of microbial additive on microbial populations, ensiling characteristics, and spoilage loss of delayed sealing silage of Napier grass

**DOI:** 10.5713/ajas.19.0471

**Published:** 2019-08-26

**Authors:** Yimin Cai, Zhumei Du, Seishi Yamasaki, Damiao Nguluve, Benedito Tinga, Felicidade Macome, Tetsuji Oya

**Affiliations:** 1 Japan International Research Center for Agricultural Sciences (JIRCAS), Tsukuba, Ibaraki 305-8686, Japan; 2 Department of Grassland Science, China Agricultural University, Beijing 100193, China; 3 Agricultural Research Institute of Mozambique, Matola 999068, Mozambique

**Keywords:** Aerobic Deterioration, Delayed Sealing, Microbial Additive, Napier Grass Silage, Quick Sealing

## Abstract

**Objective:**

To measure whether a microbial additive could effectively improve the fermentation quality of delayed-sealing (DS) silage, we studied the effects of inoculants of lactic acid bacteria (LAB) and cellulase enzyme on microbial populations, ensiling characteristics, and spoilage loss of DS silage of Napier grass in Africa.

**Methods:**

Quick-sealing (QS) and DS silages were prepared with and without LAB (*Lactobacillus plantarum*) inoculant, cellulase enzymes, and their combination. The QS material was directly chopped and packed into a bunker silo. The DS material was packed into the silo with a delay of 24 h from harvest.

**Results:**

In the QS silage, LAB was dominant in the microbial population and produced large amounts of lactic acid. When the silage was treated with LAB and cellulase, the fermentation quality was improved. In the DS silage, aerobic bacteria and yeasts were the dominant microbes and all the silages were of poor quality. The yeast and mold counts in the DS silage were high, and they increased rapidly during aerobic exposure. As a result, the DS silages spoiled faster than the QS silages upon aerobic exposure.

**Conclusion:**

DS results in poor silage fermentation and aerobic deterioration. The microbial additive improved QS silage fermentation but was not effective for DS silage.

## INTRODUCTION

Napier grass (*Pennisetum purpureum* Schumach) is the major feed in dairy cattle production systems in the tropics, including Africa [1,2]. Generally, Napier grass is widely planted because of its high biomass yield and its adaptations to survive under a wide range of soil types, fertility levels, and weather conditions [3]. The major constraint on dairying in the tropics is the shortage of feed for animal production, in terms of both quality and quantity, particularly in the dry season [4]. In Africa, the main sources of roughage for cattle are Napier grass, native grasses, and agricultural byproducts. When cattle are fed low-quality roughage, milk and meat production decrease [5,6]. Therefore, efficient utilization of local feed resources is important for promoting animal husbandry. The aim of ensiling, which is a traditional conservation method for fresh forage crops and grasses, is ensuring year-round availability of nutritious and palatable feed for livestock. To establish a forage production system to address the problem of animal feed shortages in the dry season, large-scale silage preparation technology using tropical grasses or forages has been developed and is used for local animal production.

Frequently, silage producers must weigh the consequences of delaying forage conservation against the risks of damage due to machine malfunction or natural rainfall events before harvest. The decision to delay conservation of forage in an attempt to avoid unstable weather is not without cost because it generally results in a more mature forage crop coupled with the associated reductions in nutritive value, which are widely recognized and understood [7]. Furthermore, damage to wilting forage crops or delayed sealing (DS) due to unexpected weather events or machine malfunction, which has been described as more damaging to nutritive value than delaying the harvest, are often unavoidable in the tropics [8]. Generally, DS and aerobic exposure seriously influence silage fermentation. When sealing is delayed for a long time, aerobic microbes may grow vigorously, and the substrates may be oxidized, resulting in poor fermentation during future ensiling [9]. In addition, when the silo is opened at feeding time, aerobic conditions prevail, and the silage is subject to the growth of aerobic microbes and becomes potentially unstable [10].

In the harvest season in African countries, silage preparation can be interrupted due to sudden rainfall and machine malfunction, and the grass and forage crop after harvest may sometimes experience aerobic exposure and DS. Hence, in animal feed production, it has become important to develop a means to improve silage fermentation with DS materials and inhibit aerobic deterioration. However, little information regarding silage fermentation and aerobic deterioration using DS forages in Africa is available. In addition, the question of whether microbial inoculants can improve DS silage fermentation and aerobic stability remains unclear. We examine the effects of inoculants of lactic acid bacteria (LAB) and cellulase enzymes on microbial populations, ensiling characteristics, and spoilage loss of DS Napier grass silage in Mozambique.

## MATERIALS AND METHODS

### Ensiling materials and silage preparation

Napier grass was obtained from an experimental field at the Agricultural Research Institute of Mozambique, Matola, Mozambique in February 2018. Napier grass was harvested and chopped into 1 to 2 cm lengths using a mechanical chopper (92-2S, Sida Agri-Machine Co., Ltd, Luoyang, China). The bunker silages were prepared with quick-sealing (QS) and DS forages. The QS forages were packed into a bunker silo (2×4×10 m) within 10 h of harvesting. The DS forages were interrupted by machine malfunction and rainfall, and the packing was completed approximately 24 h after harvesting. A commercial inoculant, Chikuso-1 (*Lactobacillus plantarum*, Snow Brand Seed Co., Ltd., Sapporo, Japan) was used to prepare both silages. To compare the fermentation quality between QS and DS forages and to verify the effect of microbial additives on silage fermentation, silages were also prepared in 150-L polyethylene drum can silos and subjected to the following treatments: control, LAB, cellulase, and LAB+ cellulase. The experiment was designed as a 2×4 factorial study in a completely randomized design (sealing×additive treatment) with three replicates per treatment. The LAB inoculant Chikuso-1 and Acremonium cellulase (*Acremonium cellulase*, Meiji Seika Pharma Co., Ltd, Tokyo, Japan) were used as silage additives based on the guidelines of a commercial manufacturer. The inoculant strain was originally isolated from a sealing crop that could produce large amounts of lactic acid in the silage environment. Cellulase was produced from *Acremonium cellulolyticus*, with the main components being glucanase and pectinase; the carboxymethyl cellulase activity was 7,350 U/g. The LAB was inoculated at 5 mg/kg as 1.0×10^5^ colony-forming unit (cfu)/g on a fresh matter (FM) basis. Cellulase was added at 10 mg blended with 20 mL H_2_O per kg of FM. The LAB and cellulase were diluted with deionized water, and the additive solution was sprayed using an electronic sprayer (SSP-5H, Fujiwara Sangyo Co., Ltd., Miki, Japan) to add experimental treatments. Under the control treatment, silage was sprayed with the same amount of water. The bunker and drum can silos were compacted to exclude air from forages, and a density of approximately 380 kg/m^3^ was achieved. The silos were kept at an ambient temperature of 25°C to 32°C and were opened after 60 d of ensiling to assess fermentation quality and perform a microbial analysis.

### Microbial analysis

The counts of microorganisms in the Napier grass or silages were measured by the plate count method [11]. Samples (10 g) were blended with 90 mL sterilized water and serially diluted 10^−1^ to 10^−8^ in sterilized water. The numbers of LAB were measured on Lactobacilli MRS (de Man, Rogosa, and Sharpe) agar (Difco Laboratories, Detroit, MI, USA) incubated at 30°C for 48 h under anaerobic conditions (Anaerobic Pack Rectangular Jar; 2.5 liters, Mitsubushi Gas Chemical Company INC, Tokyo, Japan). For isolation of LAB, more than 10 strains on MRS agar medium were picked randomly from each sample, and a total of 35 isolates were collected, of which 28 isolates were considered to be LAB, as determined by the Gram-stain, catalase reaction and lactic acid productivity [11]. Aerobic bacteria were counted on Nutrient agar (Nissui-Seiyaku Co., Ltd, Tokyo, Japan) incubated for 48 h at 30°C under aerobic conditions. Coliform bacteria were counted on Blue Light agar (Nissui-Seiyaku, Japan) incubated at 30°C for 48 h; the yeasts and molds were counted on Potato Dextrose agar (Nissui-Seiyaku, Japan) incubated for 48 to 72 h at 30°C. Yeasts were distinguished from molds and bacteria by colony appearance and observation of cell morphology. Colonies were counted as viable numbers of microorganisms in cfu/g of FM. For LAB identification, each colony of LAB was purified twice by streaking on a MRS agar plate. The pure cultures were grown on MRS agar at 30°C for 24 h, resuspended in a solution of nutrient broth (Difco, USA) and dimethyl sulfoxide at a ratio of 9:1, and stored as stock cultures in a deep freezer (MDF-U384, Sanyo Electric Co., Ltd, Osaka, Japan) at −80°C until further examination. The 16S rDNA sequence similarity was performed at GenBank data library by using the BLAST program as described by Cai et al [12].

### Chemical analysis

Pre-ensiled Napier grass, and their silage samples were dried in a forced air oven at 70°C for 48 h, and ground to pass a 1 mm mesh screen (FW 100, Taisite Instrument Co., Ltd, Tianjin, China) for chemical composition analyses. The data of chemical composition on dry matter (DM) basis were corrected for residual moisture after 3 h at 105°C. The DM, ash, crude protein (CP), and ether extract (EE) were analyzed by the methods 950.15, 942.05, 984.13, and 920.39 of AOAC [13], respectively. The organic matter (OM) content was calculated as the weight loss upon ashing. The neutral detergent fiber (NDF) and acid detergent fiber (ADF) were obtained according to the method of Van Soest [14] with an ANKOM A200i fiber analyzer (ANKOM Technology, Macedon, NY, USA) and were expressed exclusive of residual ash. The non-fibrous carbohydrate (NFC) according to Hall [15]. The acid detergent lignin (ADL) analysis was subsequently performed following the procedure of Van Soest [14]. The water-soluble carbohydrate (WSC) content was measured by high-performance liquid chromatography (HPLC) method as described by Cai et al [12], and the WSC was calculated as the sum of glucose, fructose and sucrose. Lactate buffer capacity (LBC) was measured by titrating with 0.1 M HCl to reduce pH from initial pH to pH 3.0 and then titrated to pH 6.0 with 0.1 M NaOH as described by McDonald et al [16].

### Fermentation analysis

The fermentation products of silage were analyzed by using cold-water extract, a 10 g wet silage sample was homogenized with 90 mL of deionized water and kept in a refrigerator at 4°C for 24 h as described by Cai [17]. Then, the material was filtered, and the filtrate was used to measure pH, ammonia nitrogen (NH_3_-N) and organic acids. The pH was measured using a glass electrode pH meter (Starter 100/B, OHAUS, Shanghai, China), the NH_3_-N analyzed by using steam distillation of the filtrates [17], the concentration of organic acid including lactic acid, acetic acid, propionic acid and butyric acid were measured by HPLC method [17] using Shodex RS Pak KC-811 column (Showa Denko K.K., Kawasaki, Japan), DAD detector (210 nm, SPD-20A, Shimadzu Co., Ltd, Kyoto, Japan), eluent (3 mmol/L HClO4, 1.0 mL/min), temperature (40°C).

### Aerobic stability measurement

After 60 d of ensiling, the bunker silos from the QS and DS silages were opened, and 5,000-g silage samples were packed into 15-L laboratory plastic silos. The silos were covered loosely, but not sealed, and then placed in a laboratory for 6 d at 26°C to 30°C. Samples from the upper surface were used to examine changes in the counts of yeast and mold, pH, lactic acid content, and temperature at 1, 3, and 6 d of aerobic incubation. The temperature was measured using a thermometer (No. 7, Ishihara-Ondokei Co., Ltd., Tokyo, Japan) at 06:00, 12:00, 18:00, and 24:00 each day, and the average temperature was used to indicate the silage temperature. The spoilage loss of silage was calculated as the percentage of the weight of molds occurring in the silage to the total weight of silage on an FM basis.

### Statistical analysis

Data on the microorganism population, chemical composition and fermentation quality after 60 d of ensiling were analyzed with a completely randomized design with a 2×4 (sealing [S] ×additive [A]) factorial treatment structure. The two ways analysis of variance (ANOVA) procedure of SAS version 9.1 (SAS Institute, Cary, NC, USA) was used for the analysis and the statistical model is as follows:

$${\text{Y}}_{\text{ijk}}=\mu +\alpha_{\text{i}}+\beta_{\text{j}}+\alpha \beta_{\text{ij}}+{ɛ}_{\text{ijk}}$$

where Y_ijk_ = observation; μ = overall mean, α_i_ = S treatment effect (i = Napier grass), β_j_ = A effect (j = 1 to 4), αβ_ij_ = S×A effect, and ɛ_ijk_ = error. The mean values were compared by Tukey’s test [18].

Data of aerobic stability on the lactic acid content, pH, temperature and counts of aerobic acid, yeast and mold after 1 d, 3 d, and 6 d of aerobic exposure were analyzed with a completely randomized design with a 2×3 (S×exposure days [D]) factorial treatment structure. The two ways ANOVA procedure of SAS version 9.1 (SAS Institute, USA) was used for the analysis.

## RESULTS

The chemical composition and microbial population of Napier grass before ensiling are shown in Table 1. The DM content of Napier grass was 29.65% for QS and 30.13% for DS. The OM, EE, ADL, and NFC contents of QS and DS were similar, ranging from 85.05% to 85.72%, 1.25% to 1.35%, 5.68% to 6.11%, and 9.54% to 11.46%, respectively, on a DM basis. The CP, NDF, and WSC contents in QS were 5.56%, 66.74%, and 3.10% of DM, respectively, higher (p≤0.01 or p = 0.03) than the corresponding values in DS. The ADF content in QS was 41.53%, which was lower (p = 0.02) than that in DS. The LBC in QS was 532.10 metabolizable energy/kg on a DM basis, which was lower (p = 0.04) than that in DS. Before ensiling, the LAB counts in the QS and DS were similar, ranging from 5.42 to 7.12 log_10_ cfu/g on a FM basis. The coliform bacterial, aerobic bacteria, yeast, and mold counts in DS were 8.85, 9.01, 6.19, and 5.90 log_10_ cfu/g of FM, respectively, and these microbes were higher than the 2 log_10_ cfu/g of FM in QS.

The identification and physiological properties of the inoculant and representative strains from Napier grass or silage are shown in Table 2. The strain Chikuso 1 was obtained from a commercial inoculant, while the representative strains NG 5, NG 7, and NG 12 were isolated from forage and silage. All strains were Gram-positive, catalase-negative rod or cocci, and homofermentative or heterofermentative bacteria. The strains NG 5 and NG 12 formed lactic acid as the D-isomer, strain NG 7 exclusively formed lactic acid as the L-isomer, and Chikuso 1 produced a racemic mixture of D- and L-lactic acid. These strains were able to grow at 15°C and fermented glucose, fructose, and sucrose. The strain Chikuso 1 grew at a lower pH condition than the others. Based on the morphological and biochemical characteristics and a 16S rRNA gene sequence analysis, these isolates were identified as *Lactobacillus plantarum*, *Weissella cibaria*, *Lactococcus lactis*, and *Leuconostoc mesenteroids*, respectively.

The chemical composition of Napier grass silages is shown in Table 3. After 60 d of ensiling, the OM, EE, and ADL contents of the QS and DS silages were similar levels at 86.26% to 88.60%, 2.14% to 2.42%, and 5.99% to 6.12% of DM, respectively. These contents in QS and DS silages did not differ markedly among the control, LAB, cellulase, and LAB+cellulase treatments. Compared to the DS silage, the QS silage had greater (p<0.05) CP and NDF contents. The CP content of the cellulase- or LAB+cellulase-treated silages were higher (p<0.05), while the NDF and ADF contents were lower (p< 0.05) than those of the control. The CP, NDF, and ADF contents were influenced (p<0.01) by S, but not the OM, EE, ADL, and NFC contents. The contents of NDF and ADF were influenced (p = 0.03) by A, but not the other chemical compositions. The interaction between S and A did not influence chemical composition.

The fermentation quality of Napier grass silage after 60 d of fermentation is shown in Table 4. When silage was prepared under QS conditions, the pH, acetic acid, propionic acid, butyric acid, and NH_3_-N (g/kg total nitrogen, TN) contents were lower (p<0.05), and the lactic acid content was higher (p< 0.05) in LAB-, cellulase-, and LAB+cellulase-treated silages than in the control. In contrast, when silage was prepared under DS conditions, all silages were of poor quality, with low lactic acid content (0.14% to 0.36% of FM), a relatively high pH (>4.20), and high NH_3_-N content (116.61 to 125.77 g/kg TN). The LAB-, cellulase-, and LAB+cellulase-treated silages were not markedly different from the control. Compared to the DS silages, the QS silages were better preserved, had higher (p<0.05) lactic acid, propionic acid, and butyric acid contents, and lower (p<0.05) pH, acetic acid, and NH_3_-N contents. The silage fermentation was influenced (p≤0.01 or p = 0.04) by S. The contents of acetic acid, propionic acid, and butyric acid were influenced (p<0.01 or p = 0.05) by A, whereas the DM, pH, lactic acid, and NH_3_-N contents were not. The lactic acid, propionic acid, and butyric acid contents were influenced (p≤0.01) by S×A, while the DM, pH, acetic acid, and NH_3_-N contents were not.

The microbial populations of Napier grass silage after 60 d of fermentation are shown in Table 5. In the QS silages, LAB were the dominant species, with counts ranging from 5.47 to 6.70 log_10_ cfu/g on an FM basis. Compared with the control silage, the counts of coliform bacteria and mold in LAB-, cellulase-, and LAB+cellulase-treated silages were significantly decreased. In the additives-treated silages, coliform bacteria and mold counts were below detectable levels (<10^2^ cfu/g on an FM basis). In the DS silage, aerobic bacteria were the dominant species, with counts ranging from 6.19 to 6.38 log_10_ cfu/g on an FM basis. The LAB, coliform bacteria, yeast, and mold counts were 4.64 to 6.16 log_10_ cfu/g of FM. The microbial population in the DS silages did not differ markedly among all treatments. The microbial population (p<0.01) were influenced by S, and the coliform bacteria counts were influenced (p<0.01) by A and S×A, whereas other microorganisms were not.

Changes in lactic acid content, yeast and mold counts, pH, and temperature during the aerobic exposure of bunker silage for 1, 3, and 6 d are shown in Table 6. In the DS silages, the yeast and mold counts in all silages increased rapidly during aerobic exposure and reached values of 9.43 and 6.93 log_10_ cfu/g on an FM basis after 6 d. With yeast and mold growth, a rise in pH and temperature and a reduction in lactic acid content were observed. In the QS silages, the yeast and mold counts, pH, and temperature were lower (p<0.05), but the lactic acid was higher (p<0.05) than in the DS silages. The lactic acid, yeasts, molds, pH, and temperature were influenced (p<0.01) by S and D. The lactic acid content, mold count, and temperature were influenced (p<0.01) by S×D; the yeast count and pH were not influenced.

**Figure 1:**
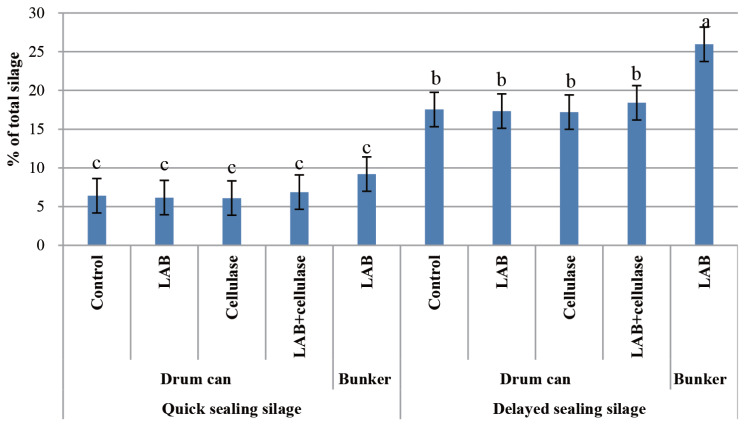
Spoilage loss of quick-sealing and delayed-sealing silage prepared with drum can and bunker silo. Data are means of three silage samples. LAB, lactic acid bacteria inoculant Chikuso-1. ^a–c^ Means±standard deviation within all the treatments with different superscript letters differ significantly from each other (p<0.05).

The spoilage loss of QS and DS silage prepared with drum can and bunker silos after 60 d of ensiling are shown in Figure 1. The spoilage losses of DS silage prepared with drum can and bunker silos were higher (p<0.05) than those for QS silage.

## DISCUSSION

Good silage fermentation depends on the moisture, WSC concentration, and buffer capacity of the forage crops or grasses [16,19,20]. In this study, the WSC content of DS Napier grass was lower than that in the QS treatment. This is consistent with the results of previous studies [21] that have clearly illustrated that respiration within hydrated plant tissues continues after mowing. According to such previous studies, the effects of DS on pre-ensiled forage result in wetting, which increases the water activity and proteolysis (as moisture stimulates plant and bacterial proteolytic enzymes), and reduces the WSC, CP, and NDF contents (due to the long aerobic exposure and the vigorous growth of epiphytic microbes). However, rainfall before ensiling may increase the moisture of DS forage, and thus the DM of DS could be similar to that of QS.

To better understand the microbial populations in Napier grass, we investigated the abundances of four kinds of microbes: LAB, aerobic bacteria, molds, and yeasts. Epiphytic LAB are naturally present in forage crops, and they are responsible for silage fermentation [12,22]. In this study, aerobic bacteria dominated the microbial populations in QS and DS. LAB were present on QS and DS in low numbers, which could not produce sufficient lactic acid during fermentation to reduce the pH and inhibit the growth of clostridia; therefore, the fermentation quality of the silage was poor. In this case, bacterial inoculants would be necessary to control the contaminating microbes during silage fermentation.

The addition of cellulase potentially increases the WSC substrate for LAB and thus may be a practical tool for enhancing silage fermentation [10]. In the present study, the contents of NDF and ADF were lower in cellulase-treated QS silages. Generally, cellulases catalyze the hydrolysis of cellulose (mainly endoglucanases, cellobiohydrolases, and β-glucosidases), and any mixture or complex of such enzymes that acts serially or synergistically can be used to decompose cellulosic material. The cellulases produced by *Acremonium cellulolyticus*, which were used in this study, contain glucanase and pectinase, indicating that this cellulase should be effective for cellulose degradation. However, the LAB or cellulase addition had no effect on the chemical composition of the DS silage. The reason may be that the abnormal growth of harmful microbes consumed sugars in the DS process, which hindered LAB growth and enzyme activity during subsequent ensiling. The microbial additive also had no significant effect on silage fermentation with the DS material.

Some epiphytic LAB, such as lactobacilli, can grow well at low pH and produce more lactic acid in the silage environment, which alters the microorganism community and silage fermentation [10,23]. In this study, there was a very low epiphytic LAB count on forage, and lactic acid-producing cocci were the main population. This is one reason why it was impossible to produce high-quality silage. Coccid LAB cannot grow long enough to produce sufficient lactic acid in a low-pH environment. When the pH of silage was higher than 4.20, the growth of clostridia was not inhibited, and butyric acid fermentation occurred. The microbial additive-treated QS Napier grass silage was of better quality than the control. Cellulase can degrade fiber, thus increasing sugar content, which can be used as a fermentation substrate by LAB. The inoculant used in this study was a homofermentative LAB, which grew well under low pH and had high lactic acid production capacity [10]. However, all of the DS silages were of poor quality, and the microbial additive did not improve the fermentation quality of the silage. In the DS Napier grass silage, the DS and prolonged exposure to air in the pre-sealing phase allowed the development of a large population of aerobic bacteria and yeast. These aerobic microbes dominated the fermentation process, leading to fermentation failure and silage deterioration. The degradation of the WSC and the fermentation acids could be attributed to the intense metabolic activity of yeasts in the early stage and molds in the later stage. Therefore, the development of aerobic microorganisms and the loss of WSC during the DS process greatly limited future silage fermentation by LAB, and a reduced delay time and QS were key to ensiling success in silage preparation.

Cai et al [10] reported that LAB improves fermentation quality but does not inhibit the growth of yeast and might increase the aerobic deterioration of silage. The deterioration of silage increases the DM loss from a silo and reduces the nutritional value of silage [10]. In addition, some aerobic microorganisms can be harmful to livestock. Therefore, preventing the aerobic deterioration of silage is very important [6,9].

Usually, acid-tolerant yeasts can survive ensiling. Thus, when silage is exposed to air after opening a silo, rapid yeast proliferation can occur [24]. In the present study, the DS silage was more susceptible to aerobic exposure than the QS silage. The aerobic bacteria, yeast, and mold counts in the DS silage were high and increased during ensiling and aerobic exposure. These microorganisms are able to grow under high pH despite being in an anaerobic environment, and they can utilize lactic acid and WSC for growth. Aerobic bacteria, yeasts, and molds were found to grow vigorously after opening of the silo, leading to rapid aerobic deterioration and spoilage loss of DS silage. Therefore, good sealing conditions not only improved silage fermentation but also reduced spoilage loss.

## CONCLUSION

The effects of LAB inoculant and cellulase enzymes on microbial populations, ensiling characteristics, and aerobic deterioration of DS silage of Napier grass in Africa were investigated. Aerobic bacteria, yeasts, and molds grew vigorously during DS and increased during ensiling and aerobic exposure, causing poor fermentation and spoilage loss of Napier grass silage. The LAB and enzyme improved fermentation quality for QS silage but were not effective for DS silage of Napier grass.

## Figures and Tables

**Table 1:** Chemical composition and microbial population of Napier grass before ensiling

Items	Napier grass		SEM	p-value

	Quick-sealing	Delayed-sealing		
Chemical composition				
DM (%)	29.65±0.97	30.13±1.12	0.61	0.64
OM (% of DM)	85.72±1.46	85.05±1.06	0.74	0.56
CP (% of DM)	5.56±0.21	5.07±0.14	0.10	0.03
EE (% of DM)	1.35±0.09	1.25±0.09	0.05	0.22
NDF (%of DM)	66.74±0.80	64.18±0.41	0.37	0.01
ADF (%of DM)	41.53±1.04	43.90±0.37	0.45	0.02
ADL (% of DM)	5.68±0.05	6.11±0.34	0.27	0.32
WSC (% of DM)	3.10±0.28	0.94±0.33	0.18	<0.01
NFC (% of DM)	11.46±1.09	9.54±0.64	0.52	0.06
LBC (ME/kg of DM)	532.10±12.59	564.30±13.20	7.45	0.04
Microbial population (log_10_ cfu/g of FM)				
Lactic acid bacteria	5.42±0.45	7.12±0.99	0.44	0.05
Coliform bacteria	6.55±0.55	8.85±0.37	0.27	<0.01
Aerobic bacteria	6.80±0.22	9.01±0.28	0.15	<0.01
Yeast	4.19±0.39	6.19±0.24	0.19	<0.01
Mold	2.78±0.71	5.90±0.09	0.29	<0.01

Data are means of three silage samples.

SEM, standard error of the mean; DM, dry matter; OM, organic matter; CP, crude protein; EE, ether extract; NDF, neutral detergent fiber; ADF, acid detergent fiber; ADL, acid detergent lignin; WSC, water-soluble carbohydrates; NFC, non-fibrous carbohydrate; LBC, lactate buffering capacity; ME, metabolizable energy; FM, fresh matter; cfu, colony-forming unit.

**Table 2:** Identification and physiological properties of inoculant strain and representative strains from Napier grass or silage

Characteristic	*Lactobacillus plantarum*	*Weissella cibaria*	*Lactococcus lactlis*	*Leuconostoc mesenteroids*
Source	Inoculant	Material	Material	Silage
Representative strain	Chikuso 1	NG 5	NG 7	NG 12
LAB characteristics				
Shape	Rod	Cocci	Cocci	Cocci
Gram stain	+	+	+	+
Catalase	−	−	−	−
Fermentation type	Homo	Hetero	Homo	Hetero
Optical form of lactate	DL	D(−)	L(+)	D(−)
Growth at				
15°C	+	+	+	+
45°C	+	−	−	−
Growth at				
pH 3.0	+	−	−	−
pH 3.5	+	−	−	−
pH 4.0	+	−	−	−
pH 4.5	+	+		+
pH 5.0	+	+	+	+
Fermentation of sugar				
Glucose	+	+	+	+
Fructose	+	+	+	+
Sucrose	+	+	+	+
Starch	w	−	−	−
16S rDNA sequence similarity with each type strain (%)	100	99.96	99.90	99.94

LAB, lactic acid bacteria inoculant Chikuso-1; +, positive; −, negative; w, weakly positive; Homo, homofermentative bacteria; Hetero, heterofermentative bacteria.

**Table 3:** Chemical composition of Napier grass silage after 60 d of fermentation

Items	OM	CP	EE	NDF	ADF	ADL	NFC

	-------------------------------------------------------------- % of DM -----------------------------------------------------------------						
Quick-sealing							
Control	87.07±1.05	4.74±0.07^b^	2.42±0.14	71.80±0.77^a^	43.03±1.06^a^	5.99±0.22	10.02±0.24^b^
LAB	88.54±0.86	5.02±0.15^ab^	2.37±0.17	71.50±0.90^a^	42.89±1.49^a^	6.05±0.18	11.56±0.28^ab^
Cellulase	88.60±1.06	5.25±0.21^a^	2.26±0.24	69.70±0.83^b^	40.49±1.01^b^	6.05±0.11	12.51±1.28^a^
LAB+cellulase	88.27±0.56	5.10±0.17^a^	2.19±0.16	69.40±0.85^b^	40.61±1.02^b^	6.08±0.18	11.63±1.01^ab^
Delayed-sealing							
Control	86.47±0.91	4.27±0.25^a^	2.26±0.21	69.93±1.28^a^	44.30±0.69^a^	6.03±0.19	10.70±1.17^a^
LAB	86.26±2.55	4.17±0.16^a^	2.15±0.17	68.53±1.41^a^	44.02±1.43^a^	6.08±0.18	10.97±1.14^a^
Cellulase	87.21±1.96	4.27±0.29^a^	2.15±0.08	68.31±1.39^a^	42.67±1.58^a^	6.12±0.23	11.01±1.06^a^
LAB+cellulase	87.86±1.51	4.15±0.09^a^	2.14±0.25	68.93±1.09^a^	42.99±2.14^a^	6.10±0.19	11.80±1.34^a^
SEM	0.83	0.11	0.11	0.63	0.79	0.11	0.59
Sealing means							
Quick	88.12±1.01	5.03±0.24^a^	2.31±0.81	70.60±1.32^a^	41.76±1.60^b^	6.04±0.15	11.43±1.17
Delay	86.95±1.70	4.22±0.19^b^	2.17±0.17	68.92±1.28^b^	43.50±1.50^a^	6.08±0.18	11.12±1.10
Additive means							
Control	86.77±0.94	4.51±0.31^b^	2.34±0.18	70.86±1.39^a^	43.66±1.06^a^	6.01±0.19	10.36±0.84^b^
LAB	87.40±2.11	4.60±0.49^ab^	2.26±0.19	70.02±1.94^ab^	43.46±1.45^ab^	6.07±0.18	11.27±0.81^ab^
Cellulase	87.91±1.60	4.76±0.58^a^	2.21±0.17	69.01±1.27^b^	41.58±1.68^c^	6.09±0.17	11.76±1.34^a^
LAB+cellulase	88.07±1.04	4.63±0.53^ab^	2.17±0.19	69.16±0.91^b^	41.80±1.99^bc^	6.09±0.17	11.71±1.06^a^
Significance of main effects and interactions							
Sealing (S)	0.07	<0.01	0.09	<0.01	<0.01	0.61	0.47
Additive (A)	0.43	0.17	0.43	0.03	0.03	0.87	0.11
S×A	0.67	0.12	0.86	0.29	0.81	0.99	0.32

Data are means of three silage samples.

OM, organic matter; CP, crude protein; EE, ether extract; NDF, neutral detergent fiber; ADF, acid detergent fiber; ADL, acid detergent lignin; NFC, non-fibrous carbohydrate. DM, dry matter; LAB, lactic acid bacteria inoculant Chikuso-1; SEM, standard error of the mean.

a–c Means±standard deviation within columns with different superscript letters differ significantly from each other (p<0.05).

**Table 4:** Fermentation quality of Napier grass silage after 60 d of fermentation

Items	DM %	pH	Lactic acid	Acetic acid	Propionic acid	Butyric acid	NH_3_-N (g/kg of TN)

	------------------------------------------------------------- % of FM ---------------------------------------------------------						
Quick-sealing							
Control	32.58±0.81	4.51±0.03^a^	0.28±0.06^c^	0.52±0.07^a^	0.09±0.03^a^	0.50±0.04^a^	80.28±17.99^a^
LAB	32.50±1.22	4.06±0.05^c^	0.74±0.09^a^	0.35±0.05^b^	0.02±0.01^b^	0.22±0.06^b^	43.88±6.57^b^
Cellulase	32.58±1.35	4.25±0.06^b^	0.56±0.06^b^	0.42±0.03^b^	0.03±0.01^b^	0.25±0.09^b^	52.37±11.62^b^
LAB+cellulase	32.49±0.89	4.03±0.04^c^	0.73±0.05^a^	0.36±0.04^b^	0.02±0.01^b^	0.23±0.04^b^	43.93±7.46^b^
Delayed-sealing							
Control	30.74±0.20	5.97±0.17^a^	0.36±0.43^a^	0.47±0.07^a^	0.02±0.01^a^	0.09±0.05^a^	125.77±21.56^a^
LAB	30.87±0.54	6.27±0.45^a^	0.15±0.08^a^	0.44±0.05^a^	0.03±0.01^a^	0.10±0.03^a^	117.06±18.92^a^
Cellulase	30.87±0.31	6.04±0.39^a^	0.14±0.07^a^	0.46±0.07^a^	0.02±0.01^a^	0.11±0.06^a^	117.78±23.76^a^
LAB+cellulase	30.64±0.13	6.05±0.28^a^	0.16±0.07^a^	0.47±0.06^a^	0.02±0.01^a^	0.10±0.03^a^	116.61±22.53^a^
SEM	0.47	0.14	0.09	0.03	0.01	0.03	10.11
Sealing means							
Quick	32.54±0.93^a^	4.21±0.20^b^	0.58±0.20^a^	0.41±0.08^b^	0.04±0.03^a^	0.30±0.13^a^	55.12±18.57^b^
Delayed	30.78±0.30^b^	6.08±0.31^a^	0.20±0.12^b^	0.46±0.05^a^	0.02±0.01^b^	0.10±0.04^b^	119.30±18.97^a^
Additive means							
Control	31.66±1.14	5.24±0.81	0.32±0.28	0.50±0.07^a^	0.06±0.04^a^	0.30±0.23^a^	103.02±0.12^a^
LAB	31.69±1.22	5.17±1.24	0.44±0.33	0.39±0.06^b^	0.02±0.01^b^	0.16±0.07^b^	80.47±22.04^a^
Cellulase	31.73±1.28	5.14±1.01	0.35±0.24	0.44±0.05^b^	0.02±0.01^b^	0.18±0.10^b^	85.08±19.54^a^
LAB+cellulase	31.56±1.16	5.04±1.12	0.45±0.31	0.42±0.08^b^	0.02±0.01^b^	0.17±0.08^b^	80.27±12.55^a^
Significance of main effects and interactions							
Sealing (S)	<0.01	<0.01	<0.01	0.04	0.01	<0.01	<0.01
Additive (A)	0.99	0.57	0.48	0.05	<0.01	<0.01	0.12
S×A	0.99	0.08	0.01	0.09	<0.01	<0.01	0.50

Data are means of three silage samples.

DM, dry matter; FM, fresh matter; NH_3_-N, ammonia nitrogen; TN, total nitrogen; LAB, lactic acid bacteria inoculant Chikuso-1; SEM, standard error of the mean.

a–c Means±standard deviation within columns with different superscript letters differ significantly from each other (p<0.05).

**Table 5:** Microbial populations of Napier grass silage after 60 d of fermentation

Items	Lactic acid bacteria	Coliform bacteria	Aerobic bacteria	Yeast	Mold

	--------------------------------------------------- Log_10_ cfu/g of FM ----------------------------------------------------------------------				
Quick-sealing					
Control	5.47±0.56^b^	3.71±1.13^a^	4.49±1.02	5.00±0.41	0.63±1.10^a^
LAB	5.99±0.43^ab^	ND	4.37±0.45	4.05±0.61	ND
Cellulase	6.70±0.20^a^	ND	4.19±0.75	3.97±0.29	ND
LAB+cellulase	6.03±0.70^ab^	ND	5.13±0.36	4.31±0.79	ND
Delayed-sealing					
Control	5.03±0.62^a^	5.22±0.58^a^	6.32±0.58	5.87±0.39	4.64±0.38^a^
LAB	5.63±0.17^a^	5.40±0.61^a^	6.38±0.48	6.16±0.73	5.20±0.56^a^
Cellulase	5.36±0.37^a^	5.32±0.52^a^	6.26±0.34	5.96±0.76	4.78±0.49^a^
LAB+cellulase	5.25±0.92^a^	5.37±0.50^a^	6.19±0.71	5.96±0.48	4.73±0.55^a^
SEM	0.32	0.32	0.36	0.34	0.30
Significance of main effects and interactions					
Sealing (S)	<0.01	<0.01	<0.01	<0.01	<0.01
Additive (A)	0.13	<0.01	0.69	0.56	0.73
S×A	0.44	<0.01	0.49	0.29	0.30

Data are means of three silage samples.

cfu, colony-forming unit; FM, fresh matter; LAB, lactic acid bacteria inoculant Chikuso-1; ND, not detected; SEM, standard error of the mean.

a,b Means±standard deviation within columns with different superscript letters differ significantly from each other (p<0.05).

**Table 6:** Changes in lactic acid content, yeast and mold counts, pH and temperature during aerobic exposure of bunker silage for 1, 3, and 6 d

Items	Lactic acid (% of FM)	Microbial population (Log_10_ cfu/g of FM)		pH	Temperature (°C)

		Yeast	Mold		
Quick-sealing					
1 d	0.52±0.05^a^	4.59±0.38^a^	ND	4.86±0.38^a^	27.21±0.38^b^
3 d	0.42±0.04^a^	5.84±0.89^a^	ND	5.26±0.27^a^	27.74±0.50^b^
6 d	0.11±0.06^b^	5.84±0.89^a^	3.80±0.23^a^	5.45±0.35^a^	30.35±1.01^a^
Delayed-sealing					
1 d	0.05±0.08^a^	7.46±0.74^b^	4.13±0.50^c^	6.30±0.85^b^	34.20±1.30^b^
3 d	ND	8.48±0.18^a^	5.30±0.39^b^	6.80±0.35^ab^	38.30±0.44^a^
6 d	ND	9.43±0.43^a^	6.93±0.74^a^	7.36±0.25^a^	35.14±0.34^b^
SEM	0.03	0.37	0.24	0.19	0.44
Significance of main effects and interactions					
Sealing (S)	<0.01	<0.01	<0.01	<0.01	<0.01
Exposure d (D)	<0.01	<0.01	<0.01	<0.01	<0.01
S×D	<0.01	0.44	<0.01	0.44	<0.01

Data are means of three silage samples.

cfu, colony-forming unit; FM, fresh matter; ND, not detected; SEM, standard error of the mean.

a–c Means±standard deviation within columns with different superscript letters differ significantly from each other (p<0.05).
